# Men want fat, women want control: sex differences in eating behaviors

**DOI:** 10.1080/15502783.2025.2550181

**Published:** 2025-09-16

**Authors:** Cassandra Evans, Jaime Tartar, Jonathan Banks, Jennifer Austin, Jose Antonio

**Affiliations:** a Nova Southeastern University, Department of Health and Human Performance, Davie, FL, USA; b Rocky Mountain University of Health Professionals, Department of Health Science, Provo UT, USA; c Nova Southeastern University, Department of Psychology and Neuroscience, Davie, FL, USA

**Keywords:** Gender, eating pattern, craving, restraint

## Abstract

**Background:**

Dietary behaviors influence an individual’s food intake and body composition. Body composition and dietary intake are related to overall health. This study investigates sex differences in the associations between dietary behaviors, food cravings, and body composition.

**Methods:**

Using a cross-sectional design, adults completed the three-factor eating questionnaire (TFEQ-R18) and food cravings (FCI) to assess dietary behaviors. Body composition was assessed using bioelectrical impedance analysis.

**Results:**

Relative to males, there were significantly higher levels of cognitive restraint and emotional eating in females. Males exhibited greater cravings for fatty foods and a higher frequency of acting on those cravings. Body fat percentage was positively correlated with emotional eating and cognitive restraint in both sexes.

**Conclusion:**

These findings suggest that sex and body composition are key factors influencing eating behavior.

